# Nickel-Fullerene Nanocomposites as Thermoelectric Materials

**DOI:** 10.3390/nano12071163

**Published:** 2022-03-31

**Authors:** Andriy Nadtochiy, Viktor Kozachenko, Oleg Korotchenkov, Viktor Schlosser

**Affiliations:** 1 Department of Physics, Taras Shevchenko National University of Kyiv, 01601 Kyiv, Ukraine; nadtku@univ.kiev.ua (A.N.); victorc@univ.kiev.ua (V.K.); 2 Department of Electronic Properties of Materials, Faculty of Physics, University of Vienna, A-1090 Wien, Austria

**Keywords:** fullerene, metal, nanocomposite, Seebeck coefficient

## Abstract

Nickel films with nanovoids filled with fullerene molecules have been fabricated. The thermoelectric properties of the nanocomposites have been measured from room temperature down to about 30 K. The main idea is that the phonon scattering can be enhanced at the C_60_/matrix heterointerface. The distribution of atoms within the Ni and Ni-C_60_ layers has been characterized by Auger depth profiling. The morphology of the grown samples has been checked using cross-sectional scanning electron microscopy (SEM). The Seebeck coefficient and electrical conductivity have been addressed employing an automatic home-built measuring system. It has been found that nanostructuring using Ar^+^ ion treatment increases the thermopower magnitude over the entire temperature range. Incorporating C_60_ into the resulting voids further increased the thermopower magnitude below ≈200 K. A maximum increase in the Seebeck coefficient has been measured up to four times in different fabricated samples. This effect is attributed to enhanced scattering of charge carriers and phonons at the Ni/C_60_ boundary.

## 1. Introduction

Fullerenes, also known as buckyballs, exist as allotropes of carbon C_60_, C_70_, etc. [1,2]. The canonical cage-like structure, C_60_, has iscosahedral symmetry with a similar electronic structure to graphene. The most effective architecture for important practical applications exploits the C_60_/matrix heterojunction [3,4]. In most electronic device applications, the active medium consists of electron-donating and -accepting regions that form a bulk network [5]. The former region is typically composed of a polymeric medium, whereas the latter is frequently done of fullerene derivatives.

Notably, the phonon scattering can be enhanced at the C_60_/matrix heterointerface [6] due to high elastic constants reported for C_60_ [7]. Obviously, this would decrease the thermal conductivity κ, which is a prerequisite of enhancement in the figure-of-merit. Further prerequisites are a high electrical conductivity σ, bearing in mind that the dimensionless figure-of-merit $ZT=\frac{S^{2}\sigma }{\kappa }T$, where T is the temperature and S is the Seebeck coefficient. In this respect, nanocomposites of highly conductive metals or degenerate semiconductors offer a novel strategy for the development of the thermoelectric materials [8,9].

Another strategy for reducing the thermal conductivity may be the use of thin films made up of metal nanoclusters [9]. In these films, the thermal conductivity is remarkably decreased due to the scattering of phonons at cluster boundaries, although the electrical conductivity is frequently governed by the tunnelling contribution to electron transport [10]. Nanocomposites made of carbon moieties such as fullerenes have excellent electric conductivity, chemical, and mechanical stability [11]. Therefore, the metal clusters in thin films can be bridged by fullerene molecules, which might introduce new functionality to the thermoelectric materials.

Consequently, there has been considerable interest over the best method to reduce the thermal conductivity and improve the thermoelectric functionality using nanocomposites and supramolecular assemblies with C_60_ [3,12,13,14,15,16,17,18,19]. Special attention has been given to metallofullerenes with metallic species trapped inside fullerene cages, which are very important to improve the performances of fullerene-based composites, particularly, in energy applications [2].

Due to high electric conductivities, metal complexes have been extensively used to achieve high performance thermoelectric materials. However, it is still quite difficult to get poly(metal-ligands) films employing solvent casting due to poor solubility of the complexes. Therefore, the thermoelectric applications of metal complexes are greatly hindered.

In this respect, materials based on single-walled carbon nanotubes (SWCNTs) have been considered as viable counterparts to metal complexes, allowing thin films to form and achieving excellent thermoelectric behavior. Recently, it has been shown that combination of metal complexes with SWCNTs could yield high thermoelectric parameters due to the strong interactions between the metal center of the complexes and π-delocalized system of SWCNTs [20].

Interestingly, in the recent experimental study of Zhou et al. [21], it has been observed that using the platinum-based complexes with SWCNTs offers high S. This innovative strategy has enhanced the power factor of SWCNTs by using a three-phase composite system. In order to enhance S, SWCNT/Pt-4 material has been introduced to the system with the highest power factor value of 471.8 μW/m K^2^ achieved in SWCNT/(Ge_0.87_Pb_0.13_Te)_0.95_(Bi_2_Te_3_)_0.05_/PtCl_4_ at 420 K. This is one of the highest values reported for p-type SWCNT/metal complex based composite films.

It is worth mentioning that an efficient novel strategy of chemical doping and side-chain cleavage has been developed to improve the thermoelectric performance of organic-based materials [22]. Thus, introducing p-type backbone of benzodithiophene (PBDTTT) with tertiary alcohol ester (TET) has resulted in enhanced power factor by up to 15 times in comparison with PBDTTT without the ester side-chains.

Noticeably, we have recently demonstrated that C_60_ molecules placed into assemblies of Ag nanoparticles enlarge the thermopower magnitude by ≈1 μV/K over the whole temperature range of that study from about 30 to 300 K [23].

In this study, we fabricate a composite nanostructured nickel–fullerene film and check the thermoelectric properties of this material. We show that embedding C_60_ molecules into nano-sized voids causes a modest increase in the value of the thermopower. The observed enhancement can most likely be attributed to a strong electron- and phonon-boundary scattering at the Ni/C_60_ interface. Our hybrid system can be used to further enhance the thermoelectric energy conversion via Seebeck coefficient grading.

## 2. Materials and Methods

**Figure 1:**
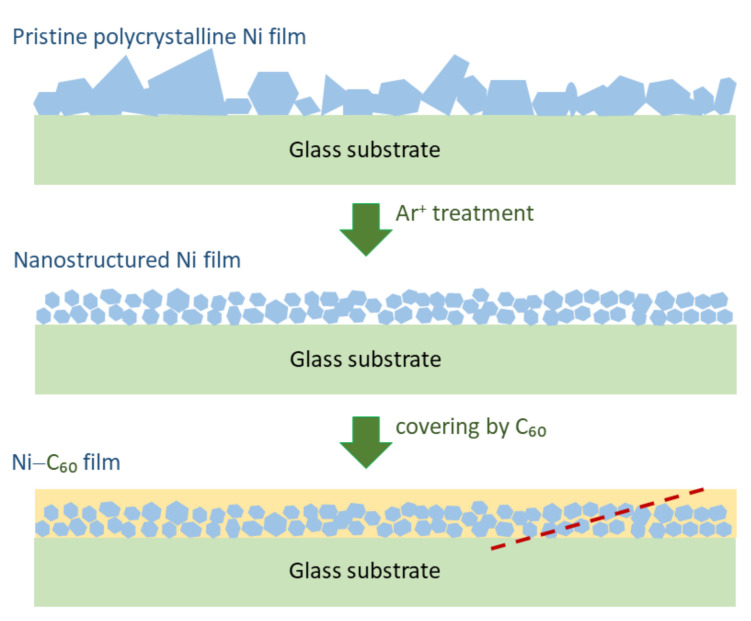
Schematics of the as-grown (**top** image), Ar^+^–treated (**middle** image) Ni film and Ni-C_60_ composite layer (**bottom** image). Dashed line in the bottom image schematically illustrate the side edge of the Ar^+^ ion etching crater used to obtain scanning electron microscopy (SEM) imagesgiven below.

Nickel-fullerene (Ni-C_60_) composite layers were grown onto glass substrates employing thermal evaporation under vacuum. Electronic or higher grade reagents were used. Glass substrates were cleaned with acetone and isopropyl alcohol before the layer deposition. The substrates were then placed in a vacuum chamber with a pressure of about 10^−5^ Torr and coated with a nickel polycrystalline film (referred to as Ni samples) having a thickness of about 20 nm, as shown in Figure 1, upper picture. The film thickness was controlled during the deposition with a quartz crystal microbalances and ex-situ by multi-angle incident ellipsometry.

Portions of the Ni samples, which can be marked as ns-Ni samples, were treated by Ar^+^ ions for 2 min. This was processed by applying DC voltage of about 1 kV between the metal anode and the Ni sample. The resulting current signal passed through the nickel film was about 1 mA. It was previously found that the metal grain sizes would shift to smaller values, which is schematically sketched in Figure 1, middle picture.

Finally, part of ns-Ni samples were covered by a C_60_ layer with a thickness of 40–50 nm forming a set of samples referred to as Ni-C_60_, as exemplified by the bottom picture in Figure 1. Using the above chamber, the 99.9%-purity C_60_ powder (SES Research) was vacuum-evaporated from a tungsten crucible heated by an electric current to 450 °C, keeping the ns-Ni sample at a temperature of about 20 °C. The sample was placed at a distance of about 15 cm from the crucible, which allowed to coat a uniform C_60_ film. The deposition rate of C_60_ was about 5 nm/s.

The distribution of atoms within the Ni and Ni-C_60_ layers was measured by Auger depth profiling. This was carried out using a JEOL Auger microprobe JAMP 9500F, Tokyo, Japan. The layer-by-layer analysis with a depth resolution of approximately 2.5 nm and in-plane averaging over ≈10 × 10 μm^2^ was done using a 1 keV Ar^+^ ion etching beam with a diameter of about 120 μm and an etching angle of several degrees. The beam scanned in ≈1 × 1 mm^2^ square raster across the film surface. Side surfaces of the etching crater (e.g., dashed line in the bottom image of Figure 1) were used to obtain cross-sectional scanning electron microscopy (SEM) images. In these measurements, the above layers of Ni and Ni-C_60_ were deposited onto conductive Si substrate.

**Figure 2:**
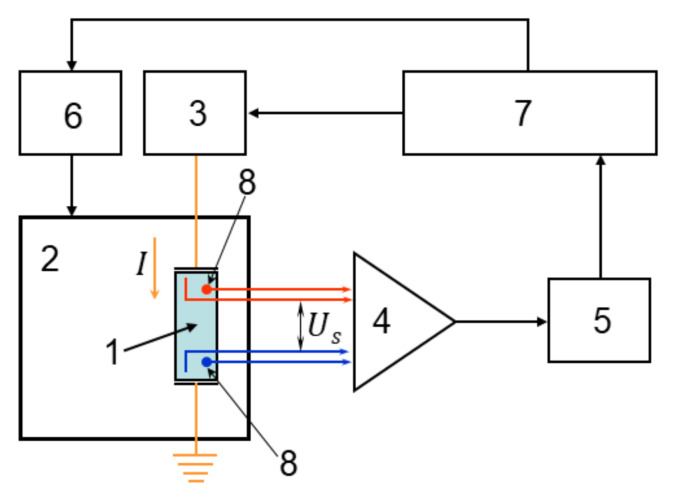
Block diagram of the automated system for the temperature-dependent Seebeck coefficient and electrical conductivity measurements. 1—sample, 2—cryostat, 3—current source, 4—switch card and amplifier, 5—analog-to-digital converter (ADC), 6—temperature controller, 7—computer, 8—temperature sensors.

The block diagram of the experimental setup for measuring the Seebeck coefficient and electrical conductivity is shown in Figure 2. Sample 1 is placed in a closed-cycle cryostat 2 (CS204, Advanced Research Systems, Macungie, PA, USA). The current source 3 consists of a 12-bit digital-to-analog converter and a buffer amplifier with a high output resistance. Copper wires are glued to the Ni layer by conductive silver paste.

The electric current I in the sample develops a voltage drop V across the layer. This voltage, amplified by a buffer amplifier 4, is sensed by a 16-bit analog-to-digital converter (ADC, AD7792, Analog Devices, Wilmington, MA, USA) 5 and feeds into a computer 7. Temperatures of the sensors 8 are changed to the voltage signals, which also feed into the computer via ADC. The cryostat temperature varied in the range from 10 to 300 K is controlled by a controller 6 (Lake Shore 331S, Lake Shore Cryotronics, Inc., Westerville, OH, USA) via the digital RS232 port of the computer. Interface program for RS 232 was created by Microsoft Visual C++ 2008, Microsoft Corporation, Redmond, WA, USA.

**Figure 3:**
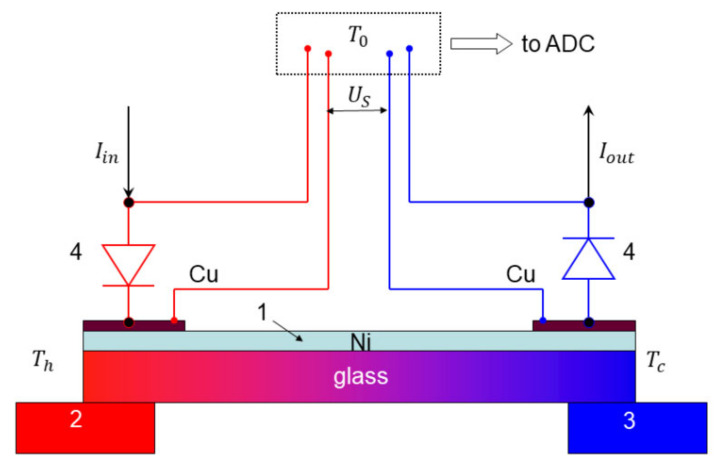
Schematics of experimental configuration for measuring Seebeck coefficient. 1—sample, 2—heater, 3—thermostat (temperature-controlled heat sink), 4—diode temperature sensors.

The Seebeck coefficient and electrical conductivity are determined in a measurement configuration shown in Figure 3. When heater 2 is powered, a temperature gradient across the sample 1 establishes. This causes the thermovoltage $U_{s}$ to appear. The sample temperature is measured by diodes 4. A small amount of thermally and electrically conductive silver paste is used to stick each of the diodes to the edge of the Ni film. When current flows through the diode, a voltage drop is formed on it, which depends almost linearly on the temperature.

The current $I_{in}$ from the current source flows through the left (“hot”) diode, then is passed through the metal film and finally goes through the second (“cold”) diode to yield $I_{out}$. Voltage drops across the two diodes (4) and thin film (1) are measured to depend upon their resistances. These voltages steer the ADC using copper wires marked as “Cu” in Figure 3, which thus implements a four-probe method of measurement. The voltage drop across the sample (1 in Figure 3) is also used to determine the film resistance.

In order to minimize the heat transfer across the film, which originates from the Joule heat generated by the diodes, the current $I_{in}$ is repeatedly switched off. After a certain time, typically 1 s, the current is switched off and $U_{s}$ slowly decreases over a certain time period, typically 9 s, as the temperature difference ΔT relaxes. Then, the current $I_{in}$ is switched on again and the cycle is repeated. Meanwhile, this Joule heat flux is rather small in comparison with a heater power. Indeed, the power supplied to the heaters is typically 100 mW, whereas the power generated by the two diodes is $P\approx \left(I_{in}+I_{out}\right)U=2I_{in}U\approx$ 0.1 mW for applied voltage U= 1 V and currents of about 0.05 mA, i.e., ≈10^3^ times smaller. Here, the diode powers add up since $I_{in}=I_{out}$.

The two diodes allow for measuring the temperatures $T_{h}$ and $T_{c}$ of the hot and cold ends of the sample, respectively. The voltage $U_{s}$ sensed by the ADC is given by the sum of the three components:

(1) $$U_{s}=S_{Cu}\left(T_{0}-T_{h}\right)+S_{Ni}\left(T_{h}-T_{c}\right)+S_{Cu}\left(T_{c}-T_{0}\right)=\left(S_{Ni}-S_{Cu}\right)\left(T_{h}-T_{c}\right),$$

where $T_{0}$ is the ambient temperature outside the cryostat. It is seen that both the magnitude and the sign of the thermovoltage depend upon the film and the wire materials.

**Figure 4:**
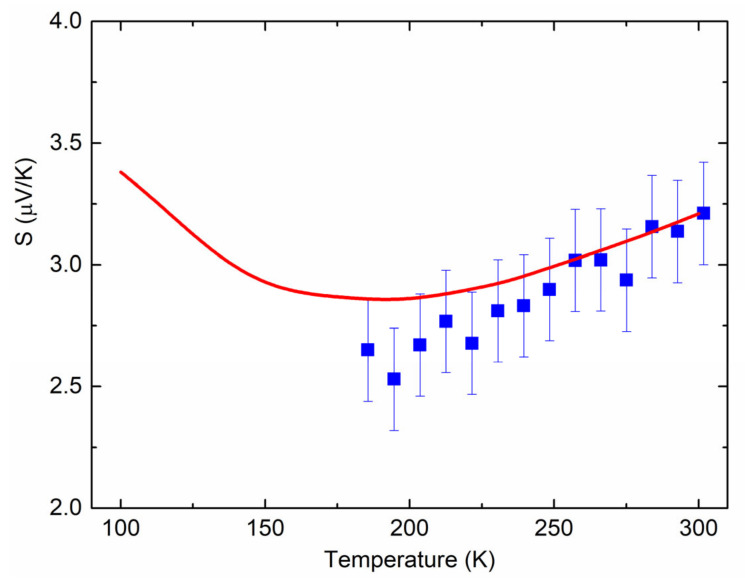
Measured temperature dependence of $S_{Cu}-S_{Al}$ (squares). Line is the difference of $S_{Cu}$ and $S_{Al}$ adapted from [25] and [24], respectively, with permissions from IOP publishing, 1958 (© IOP Publishing. Reproduced with permission. All rights reserved) and Taylor & Francis, 1977.

Therefore, in order to check the system functionality to measure Seebeck coefficient accurately, we placed, instead of sample 1 in the setup of Figure 3, a thin Al foil with well-known thermoelectric parameters [24]. The resulting measured temperature dependence $\left(S_{Cu}-S_{Al}\right)\left(T\right)$ given by squares in Figure 4 is consistent with data in the literature (line in Figure 4). Thus, for given values of $S_{Al}=-$1.6 μV/K [21] and $S_{Cu}=+$1.7 μV/K [22] at 273 K, the expected value of $\left(S_{Cu}-S_{Al}\right)=+$3.3 μV/K is in considerable agreement with the data of Figure 4 (≈+3.0 μV/K at 273 K).

## 3. Results and Discussion

**Figure 5:**
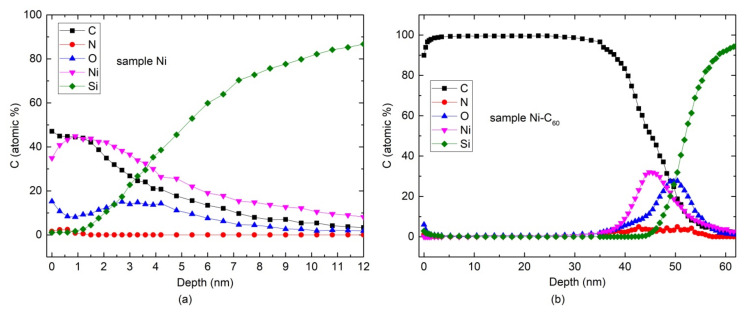
Elemental concentration as a function of depth obtained by Auger Electron Spectroscopy analysis in samples Ni (**a**) and Ni-C_60_ (**b**).

To harness enhanced phonon scattering effect without decreasing the electrical conductivity in metal composites, it is important to clarify the composition distribution inside the deposited film. In order to resolve this problem, the layer-by-layer Auger analysis is employed. Measuring the composition using successive Ar^+^ ion etching steps gives the concentration depth profiles of different elements. The principal results in Ni and Ni-C_60_ samples are shown in Figure 5a,b, respectively.

It is seen in Figure 5a that carbon atoms are incorporated into the Ni film. These C atoms may be mainly contaminated from the heater and residual gas atoms in the chamber during the deposition process. It is therefore interesting to compare the thermoelectric behavior of the carbon-contaminated Ni film and the film with incorporated fullerene molecules with the C concentration versus depth dependence displayed in Figure 5b.

**Figure 6:**
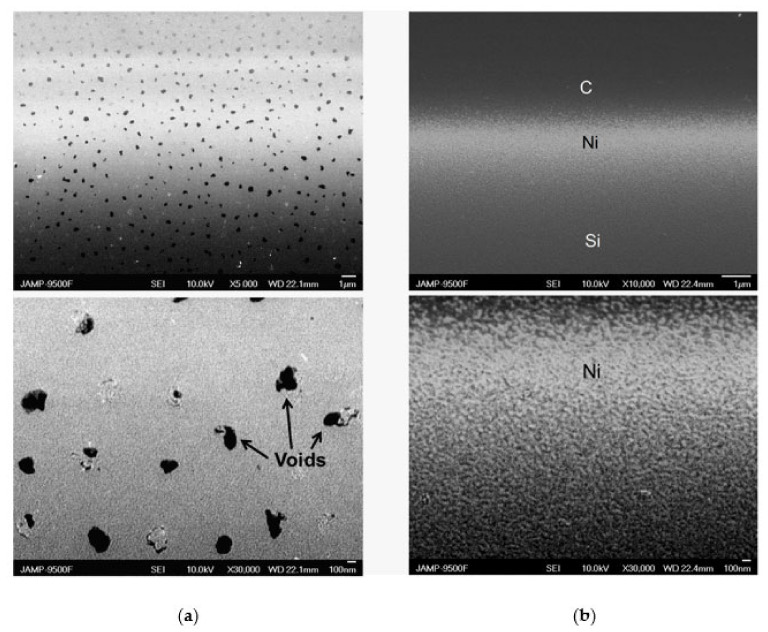
Cross-sectional SEM images of Ni (**a**) and Ni-C_60_ (**b**) samples. Lower images enlarge the middle parts of the upper images.

The cross-sectional SEM images of Ni and Ni-C_60_ samples are shown in Figure 6a,b, respectively. Upper and lower images are obtained with a smaller and greater magnification, respectively. Figure 6a illustrates the occurrence of a layer comprising a network of ~100 nm-sized voids. In clear contrast, Ni-C_60_ sample contains ~10-nm-sized carbon-filled porous network in the Ni film, which is most likely dominated by the C_60_ molecules. It is therefore quite possible that there is a layer with the mixture of C and Ni in the upper part of the lower image in Figure 6b represented by dark and bright areas, respectively. The mixing is also obvious in Figure 5b.

**Figure 7:**
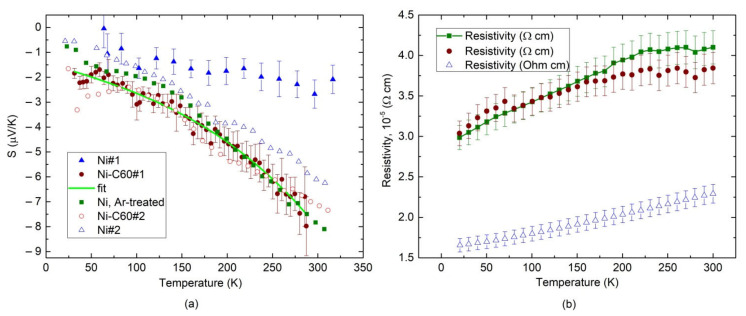
Measured temperature dependence of $S_{Ni}-S_{Cu}$ (**a**) and electrical resistivity; (**b**) in samples Ni (open and closed triangles for two different samples) and Ni-C_60_ (open and closed circles for two different samples). Squares represent the data obtained in Ar^+^–treated Ni film. Line is a fit of the closed circles to Equation (4).

This clear difference in the layer morphology is evidently meaningful to vary the thermoelectric properties of the films. The key result for the Seebeck coefficient is shown in Figure 7a. Figure 7b shows the temperature dependence of the electrical resistivity $\rho \left(T\right)$ of our samples. The $S\left(T\right)$ data of Ni sample (triangles) exhibit a negative sign and values from 1–2 μV/K to 6 μV/K in different layers. Open and closed triangles illustrate the maximum spread of the $S\left(T\right)$ dependences reproduced in our samples. A rather modest increase in the value of S is observed in the Ni-C_60_ sample (open and closed circles) with the thermopower of about 8 μV/K at around 300 K. It is also seen in Figure 7a that a phonon drag peak is seemingly observable in the Ar^+^–treated (squares) and Ni-C_60_ (circles) films between 50 and 75 K. It is also evident that incorporating C_60_ molecules increases the thermopower magnitude below ≈200 K (circles and squares in Figure 7a).

The above suggests that our deposition technique facilitates fabrication of the metal layer with different void sizes, as evident from the upper image in Figure 6a. Therefore, the issues in controlling and exploiting void scale with appropriate surface functionalization in the thermoelectric performance take on additional complexity with the use of our fabrication approach. Hence, it is difficult to obtain quantitative information on how crucial is the size of the voids in the film to affect the thermoelectric performance. Meanwhile, the experimental data follow the general trend of increasing S with decreasing the void size. As one example, nanostructuring of the Ni film using Ar^+^ ion treatment enhances S, as evidenced by the experimental data plotted in triangles and squares in Figure 7a. Moreover, the spread of the $S\left(T\right)$ dependences given by open and closed triangles in Figure 7a is in part due to varying void sizes by following the above-mentioned trend.

In bulk metals, the electron diffusion contribution to S follows a simple linear dependence on temperature [26]. This electronic contribution $S_{e}$ can be estimated using the Mott expression [26]:

(2) $$S_{e}=\frac{\pi^{2}k^{2}T}{3e}{\left(\frac{\partial ln\sigma \left(E\right)}{\partial E}\right)}_{E_{F}},$$

where k is the Boltzman constant, e is the electron charge and, in calculating the conductivity derivative, the electron energy E varies over the Fermi energy $E_{F}$. Employing useful simplifications of the functional form of the free electron relaxation time, $\tau \left(E\right)\propto E^{m}$ [27], one gets

(3) $$S_{e}=\frac{\pi^{2}k^{2}T}{3eE_{F}}\left(m+\frac{3}{2}\right).$$

Equation (3) is obtained for a free electron metal with Fermi–Dirac statistics applied to the degenerate electrons ($E_{F}\gg kT$). Beyond m=3/2 for free electrons, many interesting possibilities can be taken into account [28,29].

Electrons diffusing in the temperature gradient dissipate heat to the lattice through electron–phonon collisions, resulting in the phonon-drag thermopower $S_{ph}$ and $S=S_{e}+S_{ph}$. This is due to the fact that the electron–phonon collisions yield drag of free electrons interacting with the phonons, thus contributing to S. To contribute to $S_{ph}$, the phonon wavelengths would be greater than minimal value determined by the size of the Fermi surface. Therefore, the phonon contribution is important in metals only at low temperatures as the Fermi surface is large, while lifetimes of the short-wavelength phonons near the Fermi surface carrying heat are small [30,31]. Then, at low temperatures ($T<\theta_{D}$, the Debye temperature), $S_{ph}\propto T^{3}$, similar to the specific heat that follows the Debye $T^{3}$ law. At higher temperatures, when $T\gg \theta_{D}$ and the specific heat is saturated, the number of scattering phonons is ∝T, leading to $S_{ph}\propto 1/T$ [32].

Consequently, one expects that the Seebeck coefficient given by

(4) $$S=AT+BT^{3}$$

at low temperatures and

(5) $$S=aT+\frac{b}{T}$$

at high temperatures, where $A=\pi^{2}k^{2}/3eE_{F}$, $B=4\pi^{4}k/5e\theta_{D}$, $a=\pi^{2}k^{2}/eE_{F}$, and b is the phonon–drag coefficient [28].

Keeping in mind that $\theta_{D}$ of nickel is about 375 K [33,34], we fit the experimental S vs. temperature data to Equation (4). The lines in Figure 7a depict the fitting result for Ni and Ni-C_60_ samples.

Therefore, the scattering by phonon makes a much more significant contribution to S in Ni-C_60_ samples compared with Ni samples, which is expected from the film morphology shown in Figure 6a,b. By comparing the square and circle data points in Figure 7a, one finds that the inclusion of C_60_ into the nanostructured Ni film enhances S through electron–phonon interactions by about 35% relative change at ≈100 K. The electrical resistivity of the samples shows normal temperature behavior displayed in Figure 7b. The formation of nanovoids in Ar^+^–treated Ni film naturally increases the resistivity, as shown by squares in Figure 7b. Adding C_60_ varies the $\rho \left(T\right)$ behavior slightly, as shown by circles in Figure 7b. The low-temperature conductivity is somewhat greater in the Ni-C_60_ sample than that in the Ar^+^–treated Ni film without C_60_, as illustrated by circles and squares below ≈150 K. One therefore suggests that there is an enhanced electron scattering mechanism in the Ni–C_60_ samples, as already implied from the $S\left(T\right)$ data of Figure 7a mentioned above. Strong electron- and phonon-boundary scattering at the Ni/C_60_ interface is considered a likely mechanism for the observed enhancement.

The results of this work can be compared with the reported values of S in composite materials with carbon-based fillers. Thus, single-molecule junctions of the endohedral fullerene Sc_3_N@C_80_ connected to gold electrodes produce the mean thermopower of −2 μV/K [3]. For a single Au–C_60_–Au junction, the thermopower varies from −18 to −23 μV/K, which is among the highest values measured to date for organic materials [12]. Yee et al. reported the highest single-molecule Au/C_60_ heterojunction thermopower of −33 μV/K in comparison to an Au–Au junction thermopower of about 2 μV/K [13]. Platinum complexes composited with SWCNTs yield the values of S ranging from about 14 to 26 μV/K [21]. It is therefore seen that S≈−8 μV/K observed in our Ni/C_60_ material with ≈35% increase in S due to C_60_ can be considered to fall to a moderate value of the Seebeck coefficient.

## 4. Conclusions

In this work, we discuss the thermoelectric properties of Ni-C_60_ thin layers with a particular emphasis on the enhancement of the Seebeck coefficient due to incorporation of fullerene molecules into nanovoids made in nickel films. We also highlight the morphology of the grown films, illustrating the occurrence of a network of carbon-filled nano-sized voids. In general, incorporation of C_60_ molecules into the film increases the thermopower magnitude up to four times in different fabricated samples. This effect can be related to enhanced scattering of charge carriers and phonons at the Ni/C_60_ boundary. The results would add to our understanding of drag effects in nanostructures on the thermoelectric behavior of composite materials.

## Data Availability

Data is contained within the article.
